# Unintentional medication discrepancies at care transitions: prevalence and their impact on post-discharge emergency visits in critically ill older adults

**DOI:** 10.1186/s12877-024-05517-w

**Published:** 2024-12-18

**Authors:** Jiyoung Park, A Jeong Kim, Eun-Jung Cho, Yoon Sook Cho, Kwanghee Jun, Yoon Sun Jung, Ju-Yeun Lee

**Affiliations:** 1 College of Pharmacy and Research Institute of Pharmaceutical Sciences, Seoul National University, 1, Gwanak-ro, Gwanak-gu, Seoul, 08826 Republic of Korea; 2 Department of Pharmacy, Seoul National University Hospital, 103, Daehak-ro, Jongno-gu, Seoul, 03080 Republic of Korea; 3 College of Pharmacy, Gyeongsang National University, 501, Jinju-daero, Jinju-si, Gyeongsangnam-do, 52828 Republic of Korea; 4 Department of Critical Care Medicine, Seoul National University Hospital, 103, Daehak-ro, Jongno-gu, Seoul, 03080 Republic of Korea

**Keywords:** Unintentional medication discrepancy, Transition of care, Critically ill patient, Older adult, Chronic Disease

## Abstract

**Background:**

Unintentional medication discrepancies during care transitions pose a significant risk for medication errors, particularly in critically ill older patients. This study aimed to investigate the prevalence of such discrepancies during care transitions and their impact on post-discharge emergency department (ED) visits in this patient population.

**Methods:**

This retrospective cross-sectional study included patients aged 65 and older who were on chronic medications and admitted to the intensive care units of emergency departments (ED-ICUs) between 2019 and 2020. We evaluated unintentional medication discrepancies, including omissions or changes in medication type, dose, frequency, formulation, or administration route without clear clinical justification during care transition. The association between these discrepancies and post-discharge ED visits was analyzed using a multivariable Cox-proportional hazard model.

**Results:**

Of the 339 patients analyzed, 68% encountered unintentional medication discrepancies at some point during care transitions, with prevalence of 35% at admission, 20% during transfer, and 49% at discharge. After adjusting for confounding factors, patients with unintentional medication discrepancies had a twofold higher risk of ED visits within 30 days of discharge (HR = 2.13, 95% CI = 1.06–4.30).

**Conclusion:**

This study demonstrated a substantial prevalence of unintentional medication discrepancies among critically ill older adults during care transitions, significantly increasing the risk of ED visits within a month of discharge. The findings highlight the crucial need for systematic identification and management of medication discrepancies throughout the care transition process to enhance patient safety.

**Supplementary Information:**

The online version contains supplementary material available at 10.1186/s12877-024-05517-w.

## Introduction

Care transition, a critical process involving coordinated healthcare actions, is crucial when patients are transferred between various locations or levels of care [1]. Effective care transition is essential to avoid adverse events, care omissions or duplications, treatment delays, and consequently, increased morbidity, mortality, and healthcare costs [2]. However, the complexity of this process often leads to issues, notably medication discrepancies, which are differences between a patient's current medications and the documented regimen across different care settings [3].

Medication discrepancies can be intentional or unintentional. Unintentional discrepancies, where prescribers inadvertently alter, add, or omit medications that were prescribed before hospital admission, are of particular concern [4–7]. Unintentional medication discrepancies during transitions of care are influenced by several factors, including communication breakdown, incomplete or inaccurate documentation of patients' medication histories, patient understanding and adherence to their medication regimens, cognitive function, regimen complexity, and inadequate medication reconciliation practices [8, 9].

Patients in emergency department ICUs (ED-ICUs) are especially prone to medication discrepancies due to factors like staff fatigue, shortages, unclear instructions, and similarities in drugs [10]. Studies indicate high rates of unintentional medication discrepancies in ICU patients, both at admission and discharge. This risk is exacerbated when long-term medications are temporarily stopped in the ICU but not adequately reinstated later, potentially leading to the exacerbation of chronic conditions upon discharge [11, 12].

These discrepancies can lead to medication errors, adverse drug events (ADEs), increased healthcare costs, and higher readmission rates, particularly in older patients who often have multiple chronic conditions [13]. Older patients are particularly vulnerable to medication discrepancies because they frequently require multiple medications and undergo frequent transitions of care. This population is at high risk for numerous medication problems, including inappropriate prescribing, drug–drug interactions, drug–disease interactions and ADEs. Additionally, psychological and physiological factors in older adults may impair their ability to communicate effectively with medical and healthcare staff [14]. Prevalence of 49.5–81.9% medication discrepancies during transitions in care have been reported in this population [13, 15–17]. Therefore, older adults need to be a priority target population for research on unintentional medication discrepancies.

Recognizing the incidence of medication discrepancies and its associated outcomes is paramount for the development of strategies aimed at preventing such discrepancies. These strategies, in turn, enhancing patient safety and contribute to the reduction of healthcare costs [13]. However, there is a lack of comprehensive data on the specific impact of these discrepancies during the transition from ED-ICU to other care settings in critically ill older adults.

This study aims to fill this gap by estimating the prevalence of unintentional medication discrepancies at care transitions in critically ill older adults admitted to the ED-ICU. Additionally, this study seeks to explore the association of these discrepancies with ED visits within a month post-discharge, offering insights into potential areas for improvement in patient care and safety.

## Methods

### Study design and population

This retrospective cross-sectional study was conducted at a tertiary academic hospital. It encompassed patients aged 65 years or older who were admitted to ED-ICU and discharged from January 1, 2019, to December 31, 2020. These patients were prescribed medications for any of 22 chronic diseases. The selection of these chronic diseases was based on a prior study that identified these conditions as the most prevalent and clinically significant in older adults discharged from a tertiary hospital, ensuring relevance and consistency with existing research [4](see Supplementary Table 1).

Patients who died before discharge, who were not taking any chronic medication upon admission, who had ICU stay of less than 3 days, or who had a psychiatric disorder were excluded from the study. The last criterion is due to restrictions on accessing information on patients with psychiatric disorders within the electronic medical records of the research institution.

### Identification of unintentional medication discrepancies

Unintentional medication discrepancy was defined as medication omission or changes in the drug, dose, frequency, formula, and route of administration without any clinical explanation referring to a taxonomy of medication discrepancies and examples of clinical situations from several sources (Supplementary Table 2) [5, 7] and operational instructions provide by the MedTax [18]. MedTax, meaning medication discrepancy taxonomy, was developed to provide a common nomenclature and classification system for reporting medication discrepancies, ensuring consistent and reliable assessment.

**Fig. 1:**
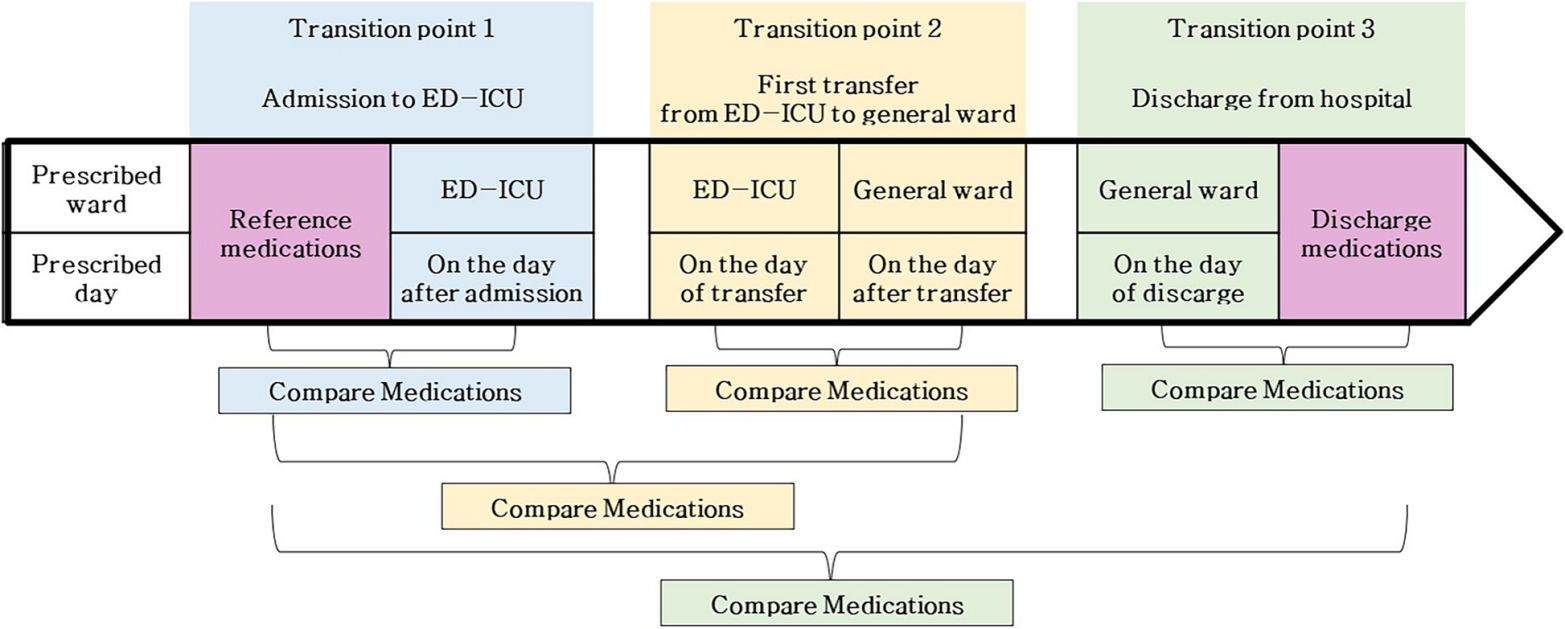
The process of detecting medication discrepancies during care transitions

The primary outcome was the prevalence of experiencing at least one unintentional medication discrepancy during the entire care transition, which included the following three transition points: (i) admission to the ED-ICU, (ii) the first transfer from the ED-ICU to a general ward, and (iii) discharge from the hospital to home or another rehabilitation hospital. With pre-admission chronic medications serving as the reference, medications prescribed at the time of admission, general ward transfer, and discharge were collected and compared at these three transition points. Additionally, medications prescribed at the time of general ward transfer were compared with those prescribed in ED-ICU on the day of transfer. At hospital discharge, discharge medications prescribed were compared with the medications prescribed in ward right before discharge, along with pre-admission medications (Fig. 1).

If there were obvious records of the reason or possible clinical explanation for discrepancy (for example, doctor's written order, hospital history, or consultation with other departments) or a possible clinical explanation for the discrepancy, it was classified as intentional and excluded. We excluded the discrepancy of medication addition for the management of condition during hospitalization. The assessment of medication discrepancy was conducted by two pharmacists including a specialist critical care pharmacist who have extensive clinical experience.

### Definition of variables

The study assessed variables to identify factors associated with unintentional medication discrepancies. These include age, sex, Charlson Comorbidity Index (CCI) score, total hospital and ICU lengths of stay, incidence and number of transfers, number of chronic medications, use of high-risk medications, chronic medication classes, and chief complaints upon admission. High-risk medications were identified using the Institute for Safe Medication Practices list of high-alert medications in community or ambulatory care settings [19]. Chronic medications were categorized based on the World Health Organization Anatomical Therapeutic Chemical (WHO-ATC) 2023 classification, and chief complaints upon admission were classified according to the International Statistical Classification of Diseases-10 coding system.

The association between unintentional medication discrepancies and post-discharge ED visits was analyzed by identifying the first ED visit within one month of discharge, including hospitalizations via the ED. This analysis adjusted for variables such as age, sex, CCI score, and lengths of stay in hospital and ICU.

### Statistical analysis

Descriptive statistics were applied to all variables, presented as median and interquartile range for quantitative variables. The prevalence of unintentional medication discrepancies during hospitalization and at each transition point was calculated. A backward stepwise multivariable logistic regression identified associative factors, including variables with a *p*-value < 0.1 in univariate analysis. Adjusted odds ratios and 95% confidence intervals were calculated. The impact of medication discrepancies on post-discharge ED visits was evaluated using a Cox proportional hazards model, adjusting for the aforementioned confounding factors. SAS® software (version 9.4) was used for all statistical analyses.

## Results

### Patients’ characteristics

**Fig. 2:**
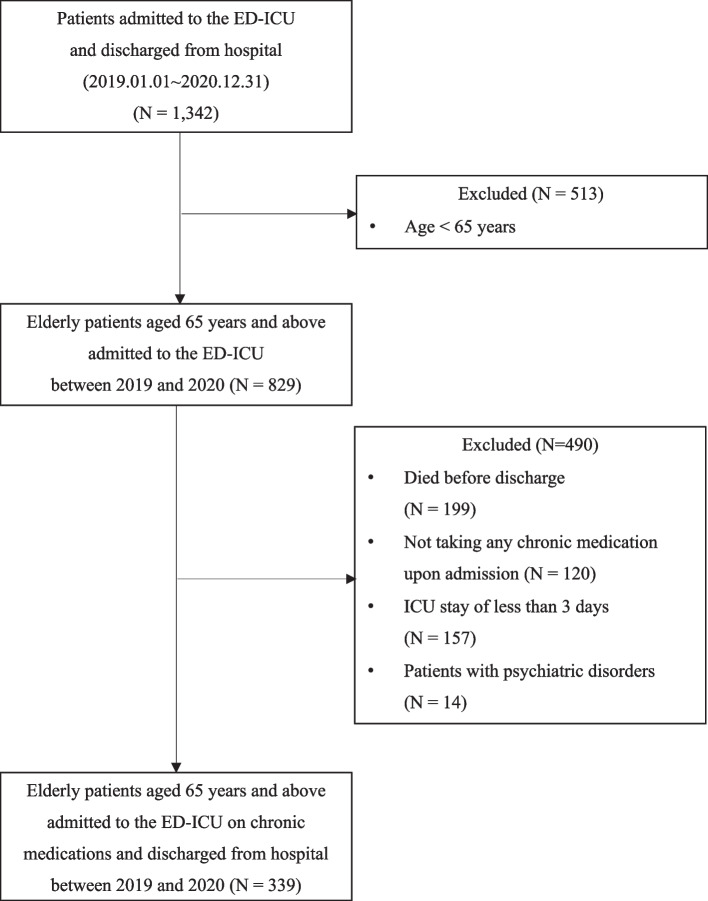
Flowchart of the patient selection process

In this study, 1,342 patients were initially considered, with 339 ultimately included in the final analysis (as illustrated in Fig. 2). The median age of these patients was 77 years. The median lengths of hospital and ICU stay were 15 days and 7 days, respectively (Table 1). The most frequently prescribed classes of chronic medications before admission were for the cardiovascular system (85%) and blood and blood-forming organs (62%). The most common chief complaint upon admission was cardiovascular disease (31%), followed by respiratory disease (27%), as detailed in Table 2.

**Table 1:** Demographics and baseline characteristics of the study population

Characteristics	No. of patients (%) (*n* = 339)
Age, years, median (IQR)	77 (71–83)
Sex	
Male	201 (59.3)
Female	138 (40.7)
Charlson Comorbidity Index, median (IQR)	1 (0–2)
≤ 1	217 (64.0)
2–3	93 (27.4)
≥ 4	29 (8.6)
Transfer during hospitalization	244 (72.0)
Number of transfers per person, median (IQR)	1 (0–1)
Number of chronic medications per person, median (IQR)	5 (3–7)
Length of stay, days, median (IQR)	15 (9–31)
Length of stay in ICU, days, median (IQR)	7 (4–15)

*IQR* Interquartile range

**Table 2:** Characteristics of preadmission chronic medication use and chief complaints on admission in the study population

Characteristics	No. of patients (%) (*n* = 339)
Use of high-risk medications	50 (14.7)
ATC classification of medication^a^	
Cardiovascular system	287 (84.7)
Blood and blood-forming organs	209 (61.7)
Alimentary tract and metabolism	143 (42.2)
Nervous system	66 (19.5)
Genitourinary system and sex hormones	61 (18.0)
Musculoskeletal system	58 (17.1)
Respiratory system	24 (7.1)
Systemic hormonal preparations excluding sex hormones and insulins	22 (6.5)
Category of chief complaints	
Cardiovascular disease	105 (31.0)
Respiratory disease	93 (27.4)
Gastrointestinal disease	34 (10.0)
Injury and poisoning	30 (8.8)
Infectious disease	20 (5.9)
Urogenital disease	16 (4.7)
Neoplasm/oncological disease	16 (4.7)
Neurological disease	16 (4.7)
Endocrine disease	9 (2.7)

^a^Numbers are not mutually exclusive. The following ATC codes were excluded: dermatologicals (ATC D), anti-infective for systemic use (ATC J), anti-neoplastic products (ATC L), insecticides and repellents (ATC P), and sensory organs (ATC S)

### Prevalence and associative factors of unintentional medication discrepancies

Throughout the care transitions, from hospital admission to ED-ICU and ultimately to discharge, a total of 951 unintentional medication discrepancies involving 654 medications were identified in 339 patients, representing 64% of the study population. At the point of admission, there were 269 unintentional medication discrepancies found in 120 patients (35%), during transfer there were 161 discrepancies in 68 patients (20%), and at discharge, 521 discrepancies were identified in 165 patients (49%). Notably, medication omissions accounted for 96% of these discrepancies.

At admission, nearly half of the medication discrepancies were found in the medication class of cardiovascular system (44%), followed by the nervous system (14%) and blood and blood-forming organs (13%). At discharge, discrepancies were detected predominantly in the medications class of cardiovascular system (45%), alimentary tract and metabolism (13%), and blood and blood-forming organs (12%). Overall, the highest occurrence of discrepancies was seen in medications for chronic cardiovascular conditions (Table 3). Specifically, lipid-modifying agents (C10), antidiabetics (A10), antithrombotics (B01), and diuretics (C03) were predominant, constituting about half of the total discrepancies (Supplementary Table 3).

**Table 3:** Types and ATC classification of medications involved in unintentional discrepancies during care transitions from admission to discharge

Type of discrepancy	ATC classification of medication	No. of medications (%)
Medication omission	C (Cardiovascular system)	292 (46.7)
	A (Alimentary tract and metabolism)	88 (14.1)
	B (Blood and blood-forming organs)	75 (12.0)
	N (Nervous system)	49 (7.8)
	G (Genitourinary system and sex hormones)	46 (7.4)
	M (Musculoskeletal system)	41 (6.6)
	R (Respiratory system)	22 (3.5)
	H (Systemic hormonal preparations, excluding sex hormones and insulins)	12 (1.9)
	Total	625 (100)
Medication change	C (Cardiovascular system)	20 (69.0)
	A (Alimentary tract and metabolism)	5 (17.2)
	B (Blood and blood-forming organs)	2 (6.9)
	M (Musculoskeletal system)	1 (3.5)
	N (Nervous system)	1 (3.5)
	Total	29 (100)

Multivariable logistic regression analysis indicated that an increased number of chronic medications (odds ratio [OR] = 1.16; 95% confidence interval [CI] = 1.05–1.28) and the use of medications for musculoskeletal system (OR = 2.88; 95% CI = 1.38–6.00) were significantly associated with a higher likelihood of medication discrepancies across all transition points (Supplementary Table 4). At each transition point in patient care, several factors were significantly associated with unintentional medication discrepancies. At admission, a higher number of chronic medications (OR = 1.12, 95% CI = 1.01–1.22) and medications for the musculoskeletal system (OR = 2.42, 95% CI = 1.35–4.35) were notable factors. During hospital transfers, the number of transfers was significantly linked to discrepancies (OR = 1.49, 95% CI = 1.14–1.95). At discharge, medications related to the genitourinary system and sex hormones were significantly associated with discrepancies (OR = 3.01, 95% CI = 1.61–5.60).

### Impact of unintentional medication discrepancies on post-discharge ed visits

Approximately 14% of patients (*n* = 49) visited the ED, including short stays and hospitalizations via ED, within one month following hospital discharge. The incidence of ED visit was notably higher in patients who experienced unintentional medication discrepancies during their care transitions, compared to those without such discrepancies (18% versus 8%, *P* = 0.014). Cox proportional hazards analysis revealed that unintentional medication discrepancies nearly doubled the risk of an ED visit within one-month post-discharge (adjusted hazard ratio [aHR] = 2.13; 95% CI = 1.06–4.30), after adjustment for various confounding factors.

## Discussion

This research highlights the significant prevalence and impact of unintentional medication discrepancies among critically ill older adults with chronic diseases in ED-ICU settings, particularly during transitions to discharge. Our findings revealed that about two-thirds of these patients experienced at least one unintentional medication discrepancy. The study also identified that the number of chronic medications at admission and the use of musculoskeletal system medications were predictors of a higher likelihood of such discrepancies.

The variation in the prevalence of unintentional medication discrepancies across studies can be attributed to factors like varying patient demographics, healthcare settings, and definitions of unintentional medication discrepancies. Our results are in line with Dong et al.’s findings, which showed a similar prevalence of discrepancies on admission among hospitalized older adults on chronic medications (32.3% vs. 35.0%) [14]. Both studies also identified cardiovascular medications as the most commonly involved class in discrepancies. Comparatively, our study reported a higher rate of discrepancies at discharge (49%) than Akram et al.'s study (23%), which did not specifically focus on older or critically ill patients [4]. This suggests that these patient groups may be more susceptible to medication discrepancies. Our analysis confirmed that a higher number of pre-admission chronic medications significantly correlated with unintentional medication discrepancies, aligning with other studies' findings [13, 15, 20]. The involvement of musculoskeletal medications in these discrepancies, as observed in our study, echoes findings by Zarif-Yeganeh et al. [20].

Crucially, our study underscored the link between unintentional medication discrepancies during hospitalization and an increased rate of ED visits within a month of discharge. This aligns with previous research indicating a significant association between medication discrepancies and post-hospital ED visits. A previous retrospective study examining the association between medication discrepancies at home and post-hospital ED visits, showed that the number of discrepancies was significantly associated with ED visits within 90 days (OR = 1.32, 95% CI 1.07–1.62), but not significantly with ED visits within 30 days of discharge [21]. A prospective study showed that medication discrepancies at the 30 days discharge increased the risk of an ED visit within 90 days of discharge significantly with having 3 or more discrepancies increase around 2.5 times versus none [22]. This underscores the importance of addressing medication discrepancies to reduce the risk of post-hospital ED visits. Improving medication management and reconciliation during transitions of care can potentially enhance patient outcomes and healthcare utilization.

Our study is pioneering in investigating unintentional medication discrepancies in critically ill older adults in South Korea, revealing both their high prevalence and detrimental clinical consequences. However, the study has limitations. First, owing to retrospective nature of the study, we had limited access to comprehensive medication histories and healthcare professionals’ prescribing intentions. Consequently, subjective judgment by the researchers may have influenced the assessment of medication discrepancies. Additionally, checking medication history retrospectively is highly susceptible to false outcomes, even with a perfectly functioning electronic database. To address this, we established operational definitions based on the taxonomy from existing papers, and experts with extensive clinical experience supervised and double-checked the process of detecting medication discrepancies. Despite these limitations, our findings align with several prospective studies using reliable real-world data, supporting the validity of our results. For example, in one prospective study, researchers used multiple information sources to collect patients' medication histories, allowing for a more precise and comprehensive medication history. They found that 42% of patients had medication discrepancies, similar to our findings of 35% [23]. Another prospective study involving patients over 60 years old, admitted to internal medicine wards, reported a 32.3% prevalence of unintentional medication discrepancies at admission, closely matching our 35% finding [14]. These similarities suggest our results are consistent with real-world data, although a prospective study design would be beneficial to mitigate the limitations of our retrospective approach.

Second, although we adjusted for some confounding factors in post-discharge ED visits, it's essential to acknowledge that not all potential confounders were fully accounted for. This may have led to an overestimation of medication discrepancies associated with ED visits. We also could not determine whether post-discharge ED visits were medication-related or not. To address this issue, future studies employing a prospective design would be advantageous.

Third, the single-center design may not fully represent nationwide practices, and therefore, there are limitations in generalizing the findings to a broader population.

Recognizing the high prevalence and potential clinical impact of medication discrepancies in this study underscores the necessity of developing and implementing effective strategies to reduce these discrepancies in critical care settings in South Korea. It is crucial to explore ways to enhance the provision of such strategies, including the activities of a multidisciplinary team that involves pharmacists, to ensure accurate medication management in the ICU setting.

Furthermore, future research should focus on identifying specific interventions and best practices that can minimize medication discrepancies. By addressing these issues, healthcare providers can improve patient safety and outcomes in critical care environments.

## Conclusion

This study sheds light on the significant occurrence of unintentional medication discrepancies in care transitions among critically ill older adults in ED-ICU settings, with these discrepancies associated with a twofold increase in post-discharge ED visits. Therefore, sufficient attention should be paid to medication discrepancies at each point of care transition during the hospitalization.

## Supplementary Information

Supplementary Material 1. Supplementary Table 1. Chronic diseases applied for inclusion criteria in this study. Supplementary Table 2. Classification of medication discrepancies. Supplementary Table 3. Types and ATC classification of medication involved in unintentional medication discrepancies at admission and discharge. Supplementary Table 4. ATC classification of medication (2nd level) and number of medications involved in unintentional medication discrepancies during care transitions from admission to discharge. Supplementary Table 5. Logistic regression analysis of associative factors for unintentional medication discrepancies.

## Data Availability

No datasets were generated or analysed during the current study.

## Abbreviations

ICU: Intensive care unit
ED: Emergency department
ED-ICU: Emergency-intensive care units
WHO: World Health Organization
ATC: Anatomical Therapeutic Chemical
