# Supplementary material for: Unintentional medication discrepancies at care transitions: prevalence and their impact on post-discharge emergency visits in critically ill older adults
Source: BMC Geriatr. 2024 Dec 18;24:1000. doi: 10.1186/s12877-024-05517-w (PMC11654400; doi:10.1186/s12877-024-05517-w)
Supplement: Supplementary file 1 — Supplementary Material 1. Supplementary Table 1. Chronic diseases applied for inclusion criteria in this study. Supplementary Table 2. Classification of medication discrepancies. Supplementary Table 3. Types and ATC classification of medication involved in unintentional medication discrepancies at admission and discharge. Supplementary Table 4. ATC classification of medication (2nd level) and number of medications involved in unintentional medication discrepancies during care transitions from admission to discharge. Supplementary Table 5. Logistic regression analysis of associative factors for unintentional medication discrepancies. [file 12877_2024_5517_MOESM1_ESM.docx]

**Supplementary Material**

**Title: Unintentional Medication Discrepancy during Care Transitions: Prevalence and their Association with Post-discharge Emergency Visits in Critically Ill Older Adults**

### List

### Supplementary Table 1. Chronic diseases applied for inclusion criteria in this study

### Supplementary Table 2. Classification of medication discrepancy

### Supplementary Table 3. ATC code and number of medications involved in unintentional medication discrepancies during care transitions from admission to discharge

### Supplementary Table 4. Logistic regression analysis of associative factors for unintentional medication discrepancies

### Supplementary Table 1. Chronic diseases applied for inclusion criteria in this study

| Asthma | Hypertension |
| --- | --- |
| Benign prostatic hyperplasia | Hyperlipidemia |
| Chronic obstructive pulmonary disease | Hyperthyroidism |
| Chronic kidney disease | Hypothyroidism |
| Congestive cardiac failure | Ischemic heart disease |
| Dementia | Liver cirrhosis |
| Depression | Osteoporosis |
| Diabetes mellitus | Parkinson’s disease |
| Dysrhythmia | Psychosis |
| Epilepsy | Rheumatoid arthritis |
| Gout | Stroke |

Adapated from Akram F, et al. Medication discrepancies and associated risk factors identified among elderly patients discharged from a tertiary hospital in Singapore. Singapore Med J 2015;56:379-84.

**Supplementary Table 2. Classification of medication discrepancy**

| 1. **No discrepancy noted.** 2. **Discrepancy is noted but no clarification is required: intentional medication discrepancy.**    1. There is an obvious record of the discrepancy (for example: physician’s written order, history of hospitalization, consultation with other departments, and history of pharmacist reconciliation).    2. There is a possible clinical explanation for the discrepancy.       1. Medication omissions   Physician’s decision not to prescribe a medication (for example: omission of antihypertensives on admission due to existing hypotension, omission of antithrombotic agents due to bleeding, withdrawal of oral medications due to preoperative fasting in the absence of parenteral formulations, Foley insertion, and ventilator application).   - - 1. Medication changes – change of dose, frequency, and route of administration.   Physician’s decision to change medication dose, frequency, or route of administration based on the patient’s clinical status (for example: adjusting the dose of hypoglycemic agent based on hypoglycemia).   - - 1. Medication changes – change of drug and formula.   A similar or alternative drug is prescribed based on the hospital’s formulary or patient’s condition upon admission.   1. **Discrepancies requiring clarification: unintentional medication discrepancy.**    1. Medication omissions   Medication prescription as per patient’s report prior to hospital admission, which is not prescribed upon admission. There is no clinical explanation for omission.   - 1. Medication changes   Change of drug, dosage, frequency, formula, and route of administration other than those as per patient’s report prior to hospital admission. The discrepancies are not explained by changes in the patient’s clinical condition, such as renal or hepatic function, nausea, and vomiting. |
| --- |

### Supplementary Table 3. ATC code and number of medications involved in unintentional medication discrepancies during care transitions from admission to discharge

| **ATC code** | **Medication class** | **No. of medications (%)** |
| --- | --- | --- |
| C10 | Lipid-modifying agents | 101 (15.4) |
| A10 | Antidiabetic drugs | 86 (13.1) |
| B01 | Antithrombotic agents | 76 (11.6) |
| C03 | Diuretics | 54 (8.3) |
| G04 | Urological agents | 46 (7.0) |
| C09 | Agents acting on the renin-angiotensin system | 45 (6.9) |
| C07 | Beta blockers | 36 (5.5) |
| C08 | Calcium channel blockers | 35 (5.4) |
| N06 | Psychoanaleptics | 33 (5.0) |
| C01 | Cardiac therapy | 27 (4.1) |
| M04 | Antigout preparations | 25 (3.8) |
| R03 | Drugs for obstructive airway diseases | 22 (3.4) |
| M01 | Anti-inflammatory and antirheumatic agents | 15 (2.3) |
| H03 | Thyroid therapy | 11 (1.7) |
| Others |  | 42 (6.0) |

### Supplementary Table 4. Logistic regression analysis of associative factors for unintentional medication discrepancies

| **Characteristics** | **Univariable analysis** | | **Multivariable logistic regression** | |
| --- | --- | --- | --- | --- |
|  | **OR (95% CI)** | ***P*-value** | **OR (95% CI)** | ***P*-value** |
| **Age (years)** |  |  |  |  |
| 65-74 | ref. |  |  |  |
| 75-84 | 1.24 (0.76-2.02) | 0.329 |  |  |
| ≥85 | 0.97 (0.50-1.85) | 0.640 |  |  |
| **Sex (female)** | 0.84 (0.54-1.32) | 0.442 |  |  |
| **Charlson Comorbidity Index** |  |  |  |  |
| ≤1 | ref. |  |  |  |
| 2-3 | 1.39 (0.83-2.34) | 0.598 |  |  |
| ≥4 | 1.40 (0.61-3.23) | 0.682 |  |  |
| **Number of chronic medications** | 1.19 (1.09-1.31) | ** < 0.01 | 1.16 (1.05-1.28) | 0.003 |
| **High-risk medications** | 1.23 (0.65-2.34) | 0.525 |  |  |
| **Length of stay** | 1.00 (0.99-1.01) | 0.828 |  |  |
| **Length of stay in ICU** | 1.01 (0.99-1.03) | 0.285 |  |  |
| **Number of transfers** | 0.84 (0.68-1.05) | 0.121 |  |  |
| **Transfer during hospitalization** | 0.99 (0.60-1.62) | 0.962 |  |  |
| **Medication classes** |  |  |  |  |
| Alimentary tract and metabolism | 1.41 (0.89-2.22) | 0.139 |  |  |
| Blood and blood-forming organs | 1.19 (0.76-1.87) | 0.455 |  |  |
| Cardiovascular system | 1.37 (0.75-2.50) | 0.303 |  |  |
| Genitourinary system and  sex hormones | 1.92 (1.02-3.61) | 0.043 | 1.749 (0.91-3.35) | 0.092 |
| Systemic hormonal preparations, excluding sex hormones and insulins | 1.22 (0.48-3.08) | 0.674 |  |  |
| Musculoskeletal system | 3.18 (1.55-6.55) | 0.002 | 2.877 (1.38-6.00) | 0.005 |
| Nervous system | 1.50 (0.83-2.69) | 0.176 |  |  |
| Respiratory system | 1.75 (0.68-4.53) | 0.250 |  |  |
| **Chief complaints upon admission** | | | | |
| Respiratory disease | 1.10 (0.67-1.81) | 0.710 |  |  |
| Gastrointestinal disease | 1.03 (0.49-2.17) | 0.929 |  |  |
| Endocrine disease | 2.00 (0.41-9.78) | 0.392 |  |  |
| Cardiovascular disease | 0.74 (0.46-1.18) | 0.203 |  |  |
| Infectious disease | 1.33 (0.50-3.56) | 0.566 |  |  |
| Neoplasm/oncological disease | 2.53 (0.71-9.05) | 0.154 |  |  |
| Neurological disease | 0.71 (0.26-1.96) | 0.509 |  |  |
| Injury and poisoning | 0.91 (0.42-2.00) | 0.820 |  |  |
| Urogenital disease | 1.25 (0.42-3.68) | 0.686 |  |  |
